# Melanocyte progenitor cells reside in human subcutaneous adipose tissue

**DOI:** 10.1371/journal.pone.0256622

**Published:** 2021-08-25

**Authors:** Yuri Ikeda, Akino Wada, Toshio Hasegawa, Mutsumi Yokota, Masato Koike, Shigaku Ikeda

**Affiliations:** 1 Department of Dermatology and Allergology, Juntendo University Graduate School of Medicine, Bunkyo-ku, Tokyo, Japan; 2 Department of Cell Biology and Neuroscience, Juntendo University Graduate School of Medicine, Bunkyo-ku, Tokyo, Japan; 3 Advanced Research Institute for Health Sciences and Juntendo University Graduate School of Medicine, Bunkyo-ku, Tokyo, Japan; 4 Atopy (Allergy) Research Center, Juntendo University Graduate School of Medicine, Bunkyo-ku, Tokyo, Japan; Università degli Studi della Campania, ITALY

## Abstract

Based on the assumption that some progenitor cells in an organ might reside in neighboring adipose tissue, we investigated whether melanocyte progenitor cells reside in human subcutaneous adipose tissue. First, we examined the expression of human melanoma black 45 (HMB45) and microphthalmia-associated transcription factor (MITF) in undifferentiated adipose-derived stem cells (ADSCs) by immunostaining, RT-PCR, and western blotting. These two markers were detected in undifferentiated ADSCs, and their expression levels were increased in differentiated ADSCs in melanocyte-specific culture medium. Other melanocytic markers (Melan A, MATP, Mel2, Mel EM, tyrosinase, KIT, and PAX3) were also detected at variable levels in undifferentiated ADSCs, and the expression of some markers was increased during differentiation into the melanocyte lineage. We further showed that ADSCs differentiated in melanocyte-specific culture medium localized in the basal layer and expressed tyrosinase and HMB45 in a 3D epidermal culture system. Melanin deposits were also induced by ultraviolet-light-B (UVB) irradiation. These results demonstrate that melanocyte progenitor cells reside in human subcutaneous adipose tissue and that these cells might have the potential to differentiate into mature melanocytes. Melanocyte and keratinocyte progenitors residing in human subcutaneous tissue can be used for the treatment of skin diseases and skin rejuvenation in the future.

## Introduction

Adipose-derived stem cells (ADSCs) are very useful in regenerative medicine because of their ease of isolation and potential to differentiate into multilineage cells. Additionally, ADSCs are easily harvested in large quantities from a small donor site.

Tissue-specific adult stem cells are heterogeneous and characterized by a mix of progenitor cells that produce cells at various stages of differentiation and ultimately terminally differentiate into different cells [1, 2].

The heterogeneity of adipose-derived mesenchymal stem cells could be caused by many different reasons that may play an important role in cell yield and growth, such as different isolation protocols, different media and culture conditions, liposuction localization, method of adipose tissue isolation, age or body mass index (BMI) [1, 3]. However, the heterogeneity of these cells can be advantageous for increasing the ability of ADSCs to differentiate [1, 4]. In addition, ADSCs have been thought to have multiple functions, and those functions could be influenced by the extracellular matrix [5] or other types of cells, such as cancer cells [6]. Moreover, ADSCs can stimulate the adhesion of melanocytes [7]. The migration and distribution of melanocyte progenitor cells is not yet known. It is known that pigment cells are formed from the neural crest and must migrate to reach their final locations [8]. After melanoblasts enter the lateral pathway, they migrate subectodermally at the same time as the dermatome undergoes an epithelial-to-mesenchymal transition into the dermis [8]. This suggests that some melanocyte progenitors could remain and reside in subcutaneous adipose tissue during embryogenesis. In fact, based on the assumption that some progenitor cells in an organ (e.g., skin) might reside in neighboring adipose tissue (e.g., subcutaneous adipose tissue), we previously identified keratinocyte progenitor cells in human subcutaneous adipose tissue [9] and found that these cells could express higher levels of type VII collagen under specific culture conditions [10]. Based on these theories and observations, we expected that there might be melanocyte progenitor cells in ADSCs.

Over 125 pigmentation-related genes have been identified previously [11]. Among the various kinds of melanocyte markers, we especially focused on MITF and HMB45 (also known as PMEL17) because MITF is thought to be a master gene for cells of the melanocyte lineage [12], and HMB45 is an antibody that recognizes melanoma- and melanocyte-specific antigens [13, 14]. HMB45 is commonly used for melanoma detection but has the added advantage that it specifically reacts with sialylated PMEL17 in the fibrillar matrix in melanosomes [11].

In this study, we further investigated whether melanocyte progenitor cells, which are reported to be maintained in the epidermis and hair follicles [15], reside in ADSCs. Towards this aim, we first evaluated nonstimulated (undifferentiated) ADSCs and differentiated ADSCs in melanocyte-specific media by various methods, including immunofluorescence microscopy, RT-PCR and immunoEM. Finally, we evaluated the expression of tyrosinase and melanin in 3D cultured differentiated ADSCs with normal human epidermal keratinocytes.

## Materials and methods

The ethical committee of Juntendo University School of Medicine suggested no requirement of ethical approval, because only commercially available cellular sources have been utilized in this study.

### Cells and culture

ADSCs (human adipose-derived stem cells; Caucasian (0000692059), Hispanic (0000421627), African (0000550179, 20TL063594), and Asian (20TL027210) #PT-5006; Lonza)) were used in this study. These ADSCs were created by Lonza, adjusting the adipose tissue obtained by liposuction in the usual way. ADSCs were cultured (passaged 2–6 times) with melanocyte medium (Dermo Life Basal Medium; Lifeline) containing PMA, b-FGF, hydrocortisone, ascorbic acid, L-glutamine, gentamicin, amphotericin B, calcium chloride, StiMel8, rh insulin, and epinephrine for 7–14 days, and these cells were designated “differentiated ADSCs”. Normal human epidermal melanocytes (NHEMs, from an infant African American CAT# KM-4009, Kurabo) and melanoma cells (G361, Caucasian, CRL-1424, ATCC) were used as positive controls.

### Immunostaining

The expression of melanocytic markers in undifferentiated ADSCs and differentiated ADSCs in melanocyte culture media was detected as follows. Undifferentiated ADSCs and differentiated ADSCs were fixed for 30 min in 4% paraformaldehyde at room temperature and blocked with PBS containing 0.1% saponin (Nacalai Tesque) and 10% goat serum (Jackson ImmunoResearch Laboratories, West Grove, PA, USA) for 1 h at room temperature followed by a 1 h incubation at 37°C with a primary antibody against human melanoma black 45 (HMB45, 1:50, Abcam Biotechnology). Samples were washed and then incubated with an Alexa Fluor 594 donkey anti-mouse IgG secondary antibody (1:300; Jackson ImmunoResearch) for 60 min at room temperature in the dark and washed three times in PBS. Nuclei were counterstained with 4’,6-diamidino-2-phenylindole (Vector Laboratories, Burlingame, CA, USA). The samples were visualized under a Keyence BZ-X700 fluorescence microscope (Osaka, Japan). Normal human epidermal melanocytes were used as a positive control.

In addition, undifferentiated and differentiated ADSCs were fixed for 30 min in 4% paraformaldehyde at room temperature and blocked with PBS containing 0.1% Triton (Wako) and 10% goat serum (Jackson ImmunoResearch Laboratories, West Grove, PA, USA) for 1 h at room temperature followed by a 1 h incubation at 37°C with primary antibodies against microphthalmia-associated transcription factor (MITF, 1:250, Abcam Biotechnology), Melan A (1:1000, Abcam Biotechnology), Mel2 (1:500, Abcam Biotechnology), MelEM (1:1; Developmental Studies Hybridoma Bank), MATP (1:400; Abcam Biotechnology), and Tyrosinase (TYR, 1:250, Abcam Biotechnology). The samples were washed and then incubated with Alexa Fluor 488 goat anti-mouse or anti-rabbit IgG (1:400; Life Technologies) and Alexa Fluor 488 donkey anti-rabbit IgG (1:500; Life Technologies) secondary antibodies for 60 min at room temperature in the dark and then washed three times in PBS. Nuclei were counterstained with 4’,6-diamidino-2-phenylindole (Vector Laboratories, Burlingame, CA, USA). The samples were visualized under a Keyence BZ-X700 fluorescence microscope (Osaka, Japan).

### Real-time PCR

The expression of melanocytic markers in undifferentiated and differentiated ADSCs was evaluated as follows. NHEMs were used as a positive control.

Total RNA was extracted from cultured cells using the RNeasy Plus Micro Kit according to the manufacturer’s protocol. A total of 2 μg RNA was converted to cDNA using the ReverTra Ace qPCR RT Kit (Toyobo, Osaka, Japan). TaqMan Master Mix (Applied Biosystems, Foster City, CA, USA) was used to amplify 1 μg cDNA for 50 cycles on a Step One Plus system (Applied Biosystems). The expression of HMB45 (PMEL TaqMan probe Hs00173854), KIT (TaqMan probe Hs00174029), MITF (TaqMan probe Hs01117294) and PAX3 (TaqMan probe Hs00240950) was normalized to that of β-actin, and the comparative cycle threshold (Ct) method using the formula 2^-ΔΔCt^ was used to calculate the relative mRNA levels.

### Small interfering RNA (siRNA)

We added HMB45 siRNA silencer select (Silencer Select gp100 s12859/s12860/s12861), Opti-MEM, and Lipofectamine RNAi MAX Reagent to ADSCs and incubated them for 24~48 h. After incubation, total RNA was extracted from the cultured cells and analyzed using the same protocol as that for real-time PCR.

### Western blotting

The expression of melanocytic markers in undifferentiated and differentiated ADSCs was assessed by western blotting. NHEM were used as a positive control.

Cells were lysed in IGEPAL Nonidet P-40 in the presence of Halt Protease and Phosphatase Inhibitor Cocktail (both from Sigma-Aldrich). Proteins were separated by 10% Tris-glycine SDS-PAGE (Bio-Rad, Hercules, CA, USA) under denaturing conditions and transferred to a nitrocellulose membrane. After blocking with 3% bovine serum albumin in Tris-buffered saline, the membrane was incubated with primary antibodies against HMB45 (1:200; Santa Cruz Biotechnology) and MITF (1 μg/ml; Abcam Biotechnology) overnight at 4°C. The blot was probed for β-actin using a monoclonal antibody (1:2000; BioLegend, San Diego, CA, USA) as a loading control. The membrane was then washed, incubated with an anti-mouse peroxidase-conjugated secondary antibody (1:1000; Santa Cruz Biotechnology and Cell Signaling Technology) and anti-rabbit antibody (1:3000; Cell Signaling Technology) at room temperature for 45 min, and developed with Luminata Forte Western horseradish peroxidase substrate (Merck Millipore, Billerica, MA, USA).

### Immunoelectron and fluorescence microscopy

Immunoelectron and fluorescence microscopy using Tokuyasu cryosections were performed as described previously with some modifications [4]. Briefly, cells were fixed with 4% paraformaldehyde buffered with 0.1 M phosphate buffer (pH 7.2) (PB) and kept overnight at 4°C. Cells were washed 3 times with PBS, then washed in 0.15% glycine in PBS and embedded in 12% gelatin in 0.1 M PB. The pellet was cut into cubic blocks of ~ 1 mm^3^ on ice, and the blocks were immersed in 2.3 M sucrose in 0.1 M PB overnight at 4°C, placed on a specimen holder (Leica Microsystems, Vienna, Austria), and quickly submerged in liquid nitrogen until use. For fluorescence microscopy, approximately 400 nm-thick sections were cut at –80°C with a Leica UC7/FC7 ultratome, picked up with a 1:1 mixture of 2% methylcellulose and 2.3 M sucrose and placed on glass slides coated with amino propyl-triethoxy silane (Matsunami Glass, Kishiwada, Japan). Thereafter, sections were incubated with a mouse anti-melanoma (1:50, Abcam, ab733, Cambridge, UK) primary antibody overnight at 4°C and further with Alexa 488-conjugated donkey anti-mouse IgG (1:200, Invitrogen Life Technologies, Carlsbad, CA) for 1 h at room temperature. Following counterstaining with 4,6-diamidino-2-phenylindole (DAPI), the sections were mounted with a coverslip and 50% glycerol in distilled water and observed by a BZ-X700 microscope (Keyence, Osaka, Japan). For immunoelectron microscopy, ultrathin cryosections (~70 nm) were cut with a Leica UC7/FC7 ultratome at approximately –120°C, picked up with a 1:1 mixture of 2% methylcellulose and 2.3 M sucrose and transferred to a nickel grid. The sections were rinsed with PBS containing 0.15% glycine, treated with 1% BSA in PBS, and incubated overnight at 4°C with a mouse anti-melanoma antibody (1:5, Abcam, ab733, Cambridge, UK), followed by incubation with donkey anti-mouse IgG conjugated to 12 nm colloidal gold particles (1:40, The Jackson Laboratory, Bar Harbor, ME). The sections were then fixed with 1% GA in PBS, embedded in a thin layer of 1.8% methylcellulose with 0.4% uranyl acetate (pH 4.0), air-dried, and observed with a Hitachi HT7700 electron microscope (Hitachi, Tokyo, Japan).

### Mel2 sorting

Mel2-positive and Mel2-negative undifferentiated ADSCs were sorted by using AutoMACS Pro (Ver.4.2.3 Miltenyi Biotec). First, undifferentiated ADSCs digested with 0.25% trypsin EDTA were centrifuged and then washed with 1% BSA/PBS. These cells were incubated with an anti-Mel2 antibody as the primary antibody and then washed again with 1% BSA/PBS. The cells were sorted by AutoMACS in positive selection mode and “pulsed” with micro MACS beads coated with an anti-mouse IgG antibody as the secondary antibody. The expression of melanocytic markers in Mel2-positive and Mel2-negative cells was evaluated by using the same protocols as those for real-time PCR and western blot.

### The 3,4-dihydroxy-L-phenylalanine (L-DOPA) reaction assay

Undifferentiated ADSCs, differentiated ADSCs, NHEMs, and Mel2-positive and Mel2-negative cells were washed with PBS and fixed with methanol at room temperature for 20 minutes. Then, these cells were incubated at 37°C with 5 mM L-dopa (Sigma Aldrich) in 0.1 M sodium buffer (pH 6.8) for 30 hours in the dark. After being washed with PBS 3 times, the cells were photographed under a light microscope.

### Three-dimensional cultured skin models

We constructed three-dimensional (3D) cultured skin models using normal human epidermal keratinocytes (NHEKs; cat #KK-4009, Kurabo), normal human epidermal melanocytes (NHEM) and differentiated ADSCs in melanocyte culture media. A gel layer comprised of collagen type 1 (liquid A:B:C = 8:1:1, Collagen gel culturing kit, Cellmatrix TypeⅠ-A, Nitta Gelatin, Osaka, Japan), collagen type 4 (1:600, Cellmatrix TypeⅣ, Nitta Gelatin, Osaka, Japan), and normal human dermal fibroblasts (NHDFs; 30-60years old man, primary culture) was created to mimic the dermis. Then, NHEKs and NHEMs or NHEKs and differentiated ADSCs were seeded on the gel 5 days after creation of the gel layer. Three days later, NHEKs were added to the gel. Five days later, the surface medium was removed, and the cells were exposed to air for 6 days.

For the induction of melanin deposits, we irradiated the gel with 15 mJ/well UVB [16], and then the cells were fixed with 4% PFA and embedded in paraffin after an additional 48 h of culture. Fontana-Masson staining was used to confirm melanin deposits.

After deparaffinization and activation, the tissues were blocked with normal goat serum (1:200; Vector Laboratories) diluted with bovine serum albumin. The tissues were incubated with primary antibodies against HMB45 (1:25; Abcam Biotechnology) and TYR (1:100; Abcam Biotechnology) overnight at 4°C. After washing with PBS, endogenous peroxidases were blocked with 0.3% H_2_O_2_/methanol for 30 min. The tissues were then washed, incubated with a goat anti-mouse biotin IgG secondary antibody (1:300; Molecular Probe) at room temperature for 60 min, and treated with avidin (1:300; Dako) for another 60 min. The cells were stained with DAB (50 ml PBS+500 μl DAB+ 10 μl hydrogen peroxide) for 5 min, nuclei were stained with hematoxylin for 1 min, and then the cells were washed for 5 min. The tissue was sealed after dehydration with alcohol and xylene.

## Results

Various types of ADSCs from different ethnicities were utilized. All ADSCs were purchased from different companies (details are mentioned in the Methods section). Most ADSCs analyzed in detail were of Caucasian origin. Normal human epidermal melanocytes were used as positive controls.

### The expression of melanocytic markers in undifferentiated and differentiated ADSCs detected by immunofluorescence microscopy

**Fig 1:**
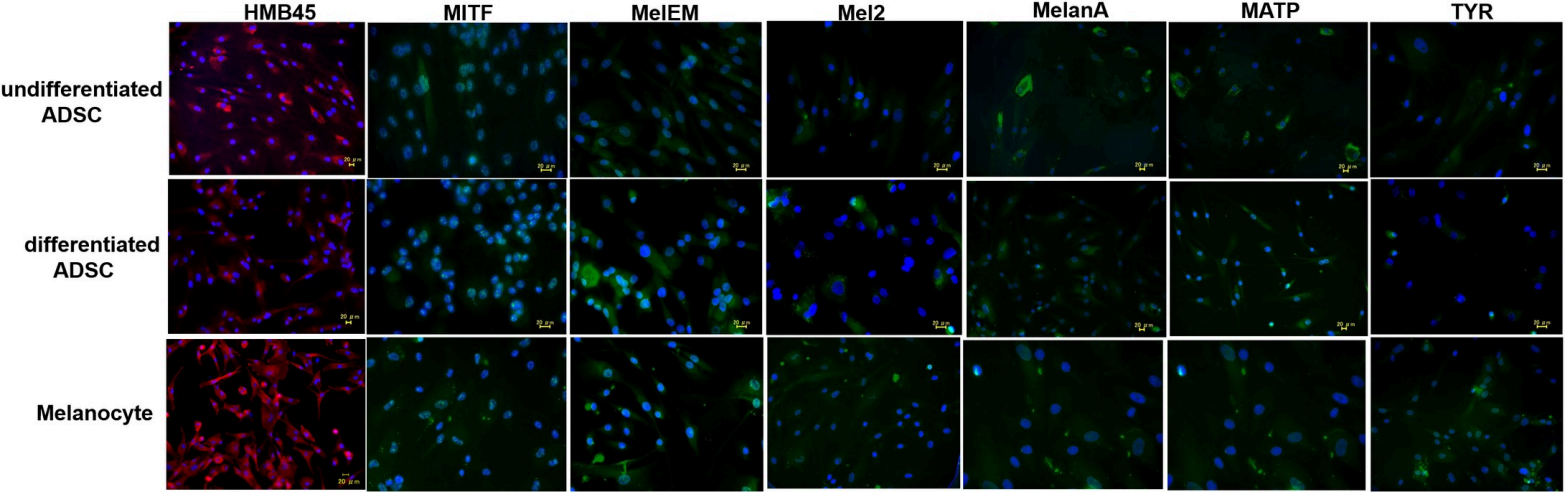
The expression of melanocytic markers in undifferentiated and differentiated ADSCs detected by immunofluorescence microscopy. The expression levels of HMB45, MITF, MelEM, Mel2, MelanA, MATP, and tyrosinase (TYR) in undifferentiated and differentiated ADSCs, as well as NHEMs, were examined by immunofluorescence microscopy. All melanocytic markers except for TYR were detected.

The expression of HMB45 and MITF was observed in undifferentiated ADSCs and melanocyte-specific media-differentiated ADSCs by immunofluorescence microscopy (Fig 1, S1A and S1B Fig in S1 File). The expression of other melanocytic markers (MelEM, Mel2, MelanA, MATP) except for TYR was also shown by immunofluorescence microscopy (Fig 1, S1A and S1B Fig in S1 File). The abovementioned expression patterns were similarly confirmed in ADSCs from other ethnic origins (African and Asian) (S1A and S1B Fig in S1 File).

### The expression of melanocytic markers in undifferentiated and differentiated ADSCs detected by RT-PCR

**Fig 2:**
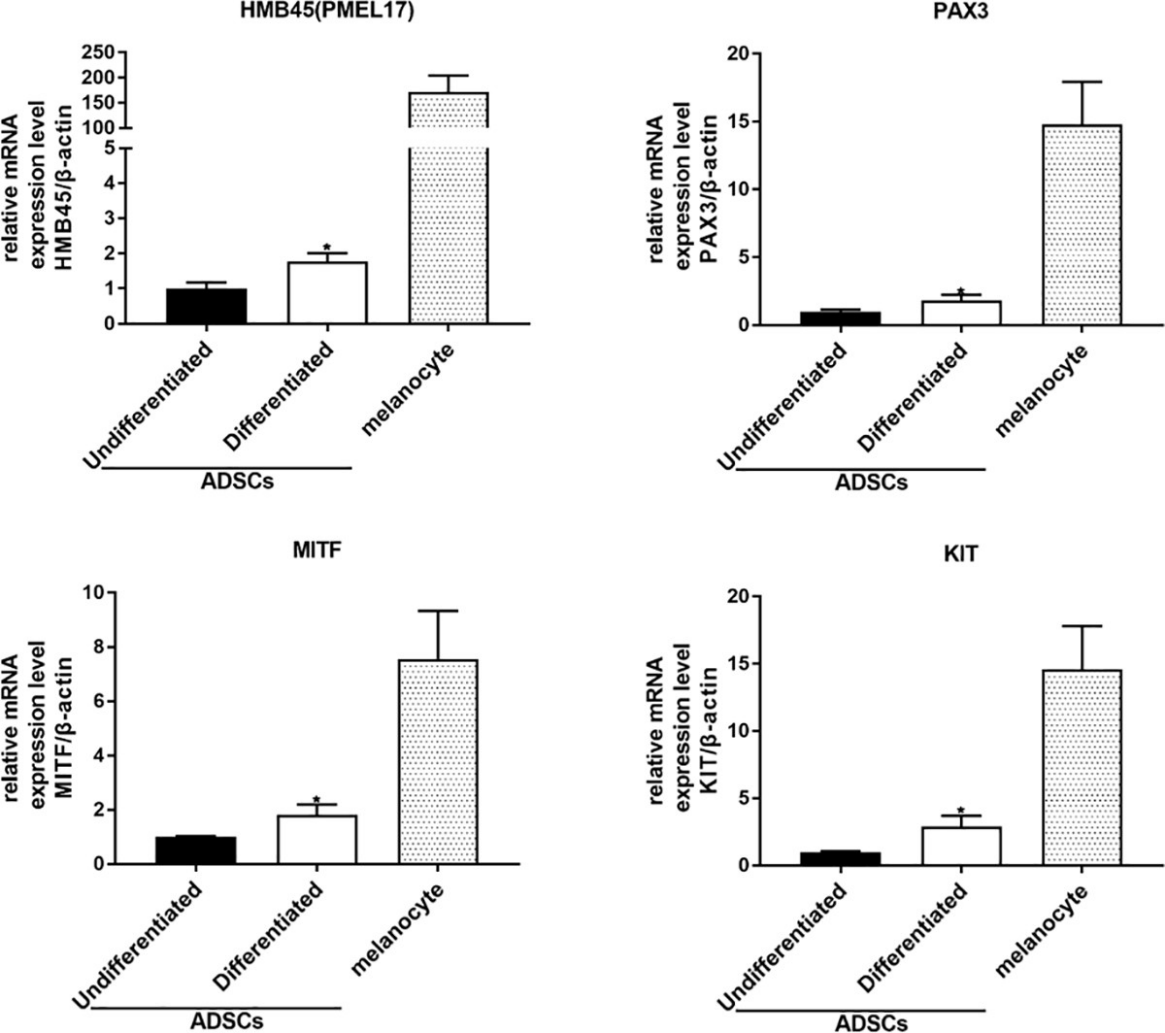
The expression of melanocytic markers in undifferentiated and differentiated ADSCs detected by RT-PCR. The expression levels of HMB45, MITF, PAX3, and KIT in undifferentiated ADSCs and differentiated ADSCs were compared with those in melanocytes by RT-PCR. The expression levels of HMB45, MITF, PAX3, and KIT were higher in differentiated ADSCs than in undifferentiated ADSCs.

The expression levels of HMB45, MITF, PAX3, and KIT in undifferentiated ADSCs and differentiated ADSCs were compared with those in melanocytes by RT-PCR. The expression levels of HMB45, MITF, PAX3, and KIT were higher in differentiated ADSCs than in undifferentiated ADSCs (Fig 2, S2A and S2B Fig in S1 File).

The abovementioned expression patterns were similarly confirmed in ADSCs from other ethnic origins (African and Asian) (S2A and S2B Fig in S1 File).

### siRNA-mediated downregulation of HMB45 in differentiated ADSCs

**Fig 3:**
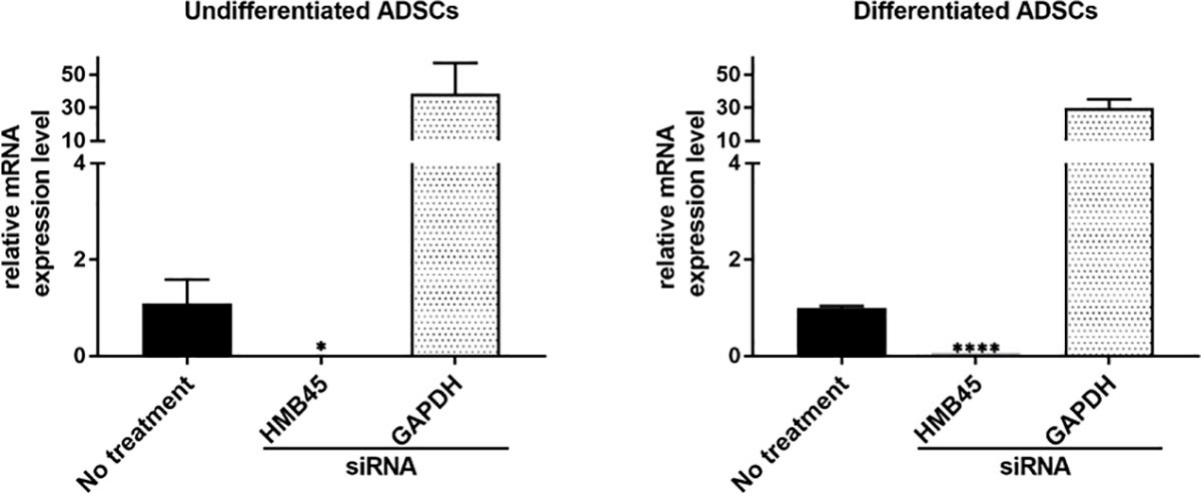
siRNA-mediated downregulation of HMB45 in differentiated ADSCs. siRNA resulted in the downregulation of HMB45 in undifferentiated ADSCs and differentiated ADSCs.

The constitutive expression of HMB45 in undifferentiated ADSCs was completely inhibited by siRNA (Fig 3, S3A and S3B Fig in S1 File).

The abovementioned expression patterns were similarly confirmed in ADSCs from other ethnic origins (African and Asian) (S3A and S3B Fig in S1 File).

### The expression of HMB45 and MITF in undifferentiated and differentiated ADSCs by western blotting

**Fig 4:**
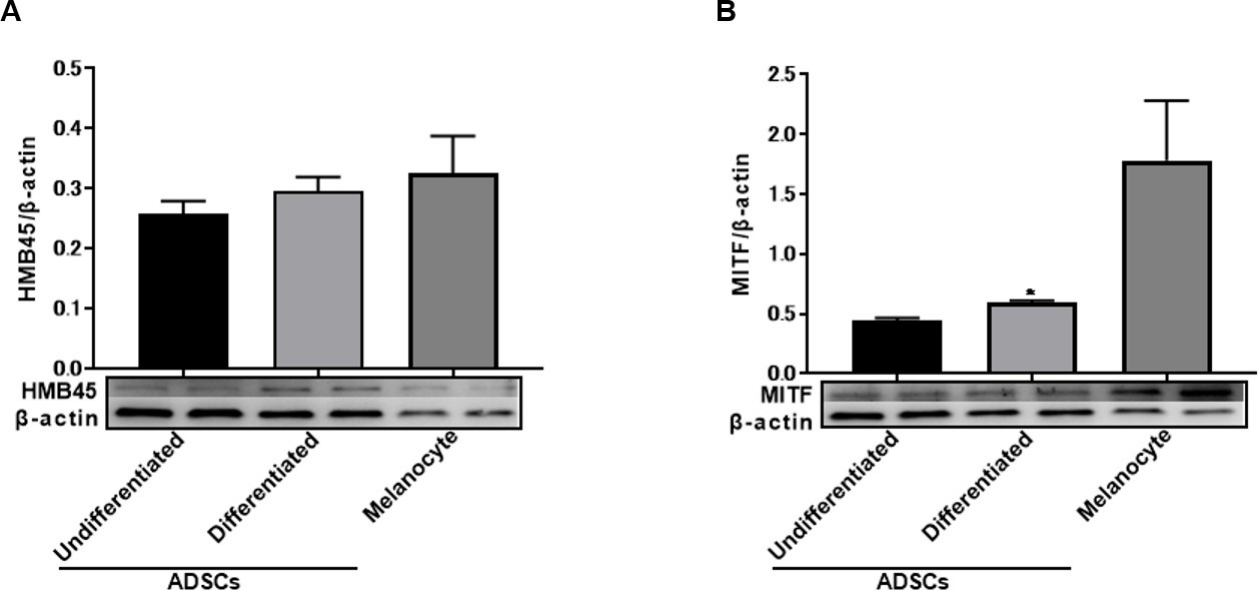
The expression of HMB45 and MITF in undifferentiated and differentiated ADSCs by western blotting. HMB45 expression in undifferentiated and differentiated ADSCs was detected by western blotting (A). MITF expression in undifferentiated and differentiated ADSCs was detected by western blotting (B). The expression of MITF was increased in differentiated ADSCs.

HMB45 and MITF expression in undifferentiated and differentiated ADSCs was detected by western blotting (Fig 4A and 4B). The expression of MITF was increased in differentiated ADSCs (Fig 4B).

### Immunofluorescence and immunoelectron microscopy with an anti-HMB45 antibody in undifferentiated ADSCs, differentiated ADSCs and melanoma cells

**Fig 5:**
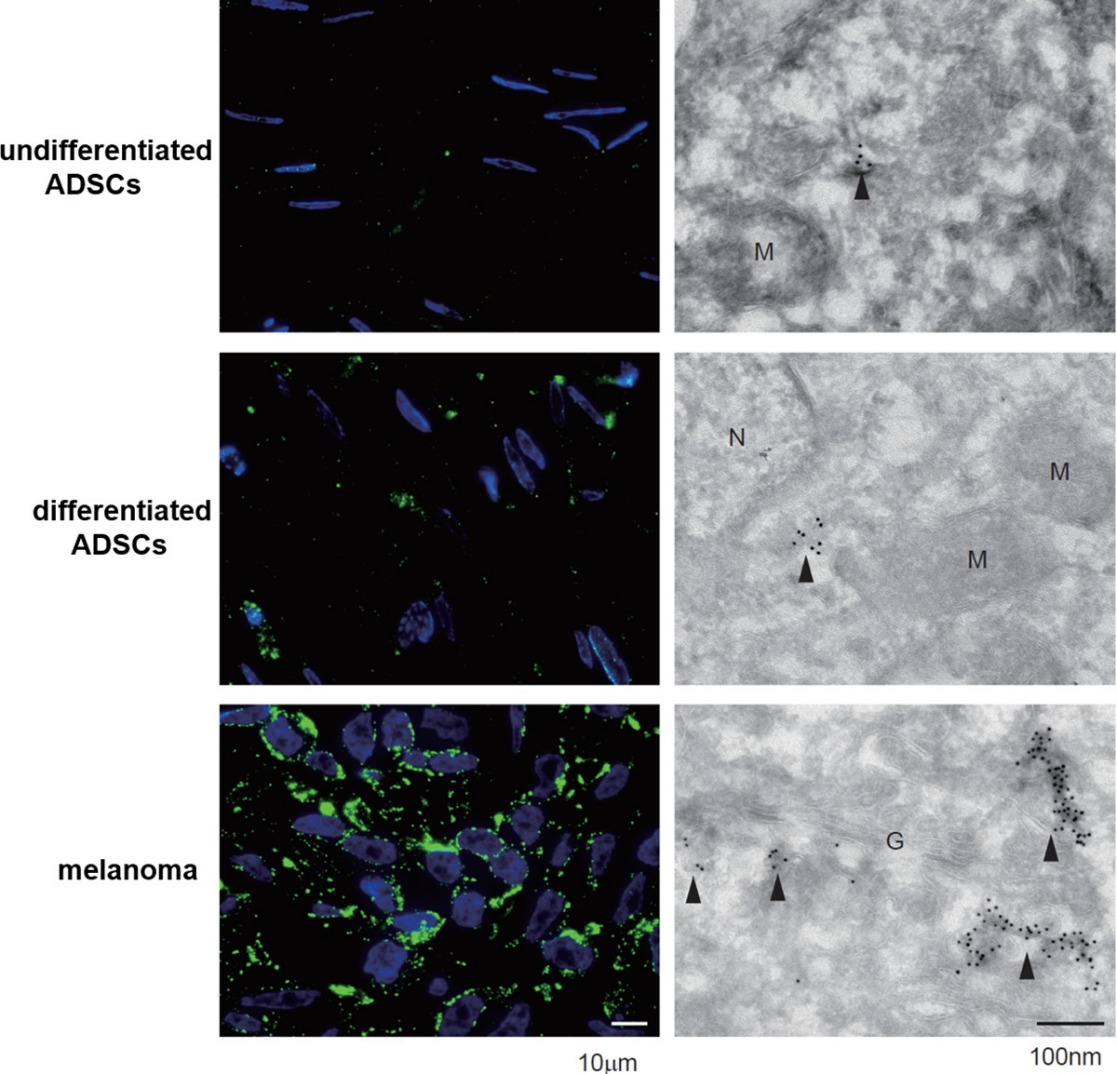
Immunofluorescence and immunoelectron microscopy with an anti-HMB45 antibody in undifferentiated ADSCs, differentiated ADSCs and melanoma cells. Note that the immunostaining intensity was increased in differentiated ADSCs. Immunogold particles mainly reacted with tubular structures due to the lack of mature melanosomes.

Note that the immunostaining intensity was increased in differentiated ADSCs. Immunogold particles mainly reacted with tubular structures due to the lack of mature melanosomes (Fig 5).

### Mel2-positive cells highly express melanocytic markers

We sorted ADSCs with Mel2, which is a melanocytic membrane protein marker.

**Fig 6:**
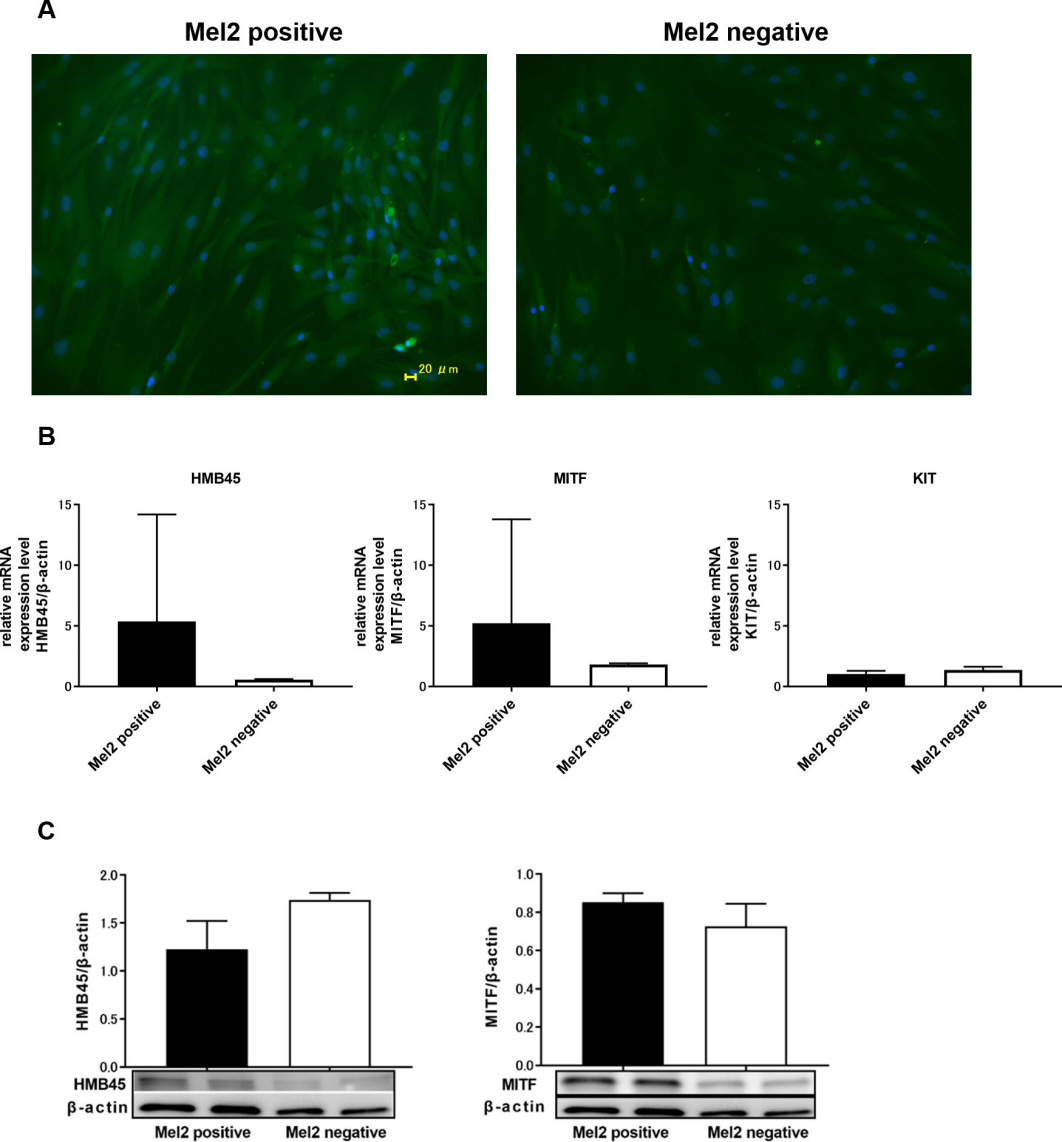
The expression of HMB45 and MITF in Mel2-positive and Mel2-negative cells. The expression of HMB45 and MITF in Mel2-positive cells was increased compared to that in Mel2-negative cells, as shown by immunofluorescence microscopy (A). The expression of HMB45 and MITF in Mel2-positive cells was increased compared to that in Mel2-negative cells, as shown by RT-PCR (B). There was almost no difference in the expression of KIT in Mel2-positive cells and Mel2-negative cells, as shown by RT-PCR (B). The expression of MITF in Mel2-positive cells was increased compared to that in Mel2-negative cells, as shown by western blot analysis (C).

Mel2-positive cells highly expressed melanocytic markers (HMB45 and MITF) according to immunofluorescence microscopy (Fig 6A), RT-PCR (Fig 6B), and western blot (Fig 6C). There was almost no difference in the expression of KIT in Mel2 positive cells and Mel2 negative cells according to RT-PCR (Fig 6B).

### Pigmented cells were detected among differentiated ADSCs in an L-DOPA reaction assay

**Fig 7:**
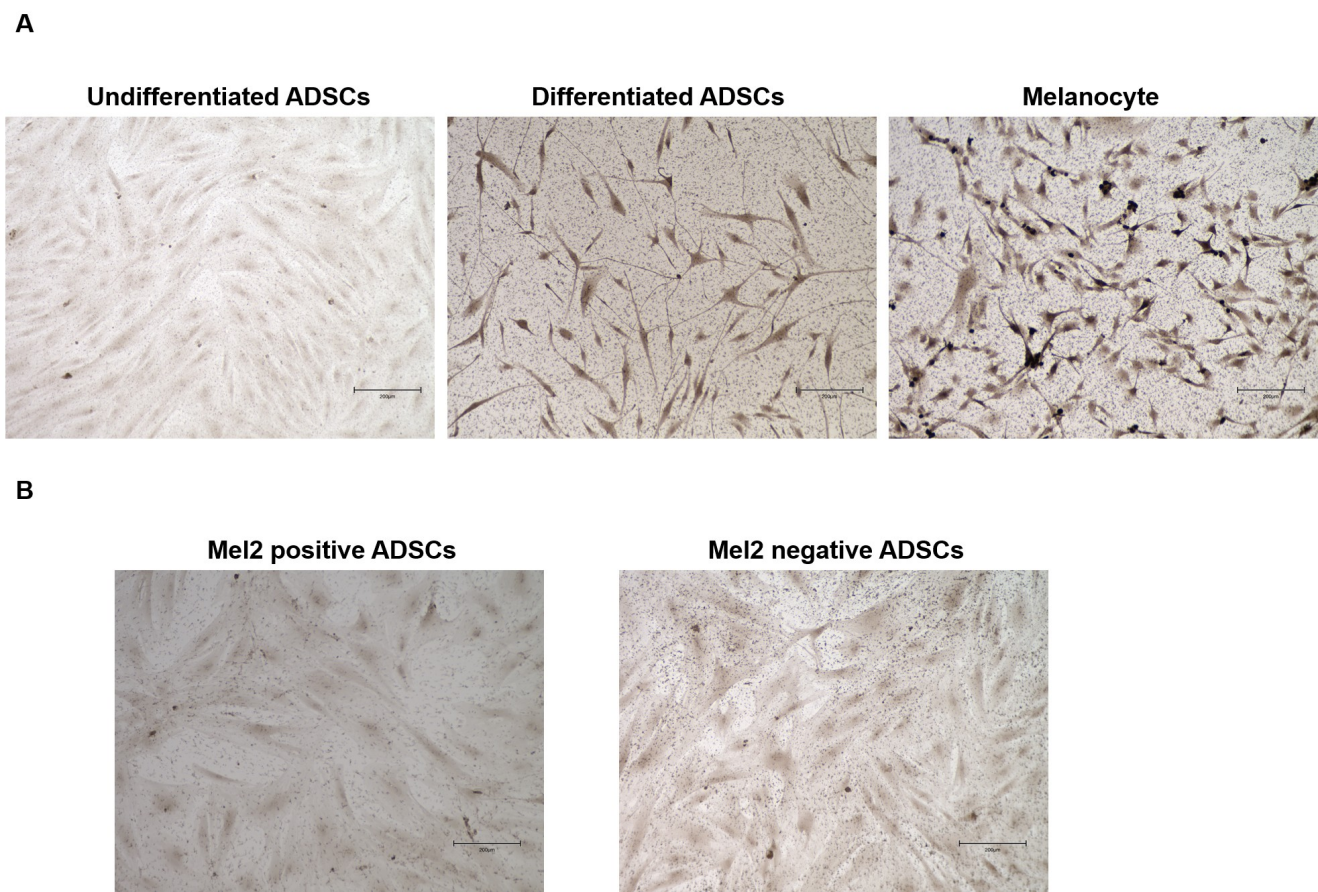
Pigmented cells were detected among differentiated ADSCs in an L-DOPA reaction assay. An L-DOPA reaction assay was performed on undifferentiated ADSCs, differentiated ADSCs, NHEMs, and Mel2-positive and Mel2-negative cells. Pigmented L-DOPA-positive cells were observed among differentiated ADSCs and NHEMs (A). The L-DOPA reaction was not significantly different in Mel2-positive cells and Mel2-negative cells (B).

An L-DOPA reaction assay was performed on undifferentiated ADSCs, differentiated ADSCs, NHEMs, and Mel2-positive and Mel2-negative cells. Pigmented L-DOPA-positive cells were observed among differentiated ADSCs and NHEMs (Fig 7A). The L-DOPA reaction was not significantly different in Mel2-positive cells and Mel2-negative cells (Fig 7B).

### Three-dimensional epidermal model by NHEKs + NHEMs or NHEKs + differentiated ADSCs

**Fig 8:**
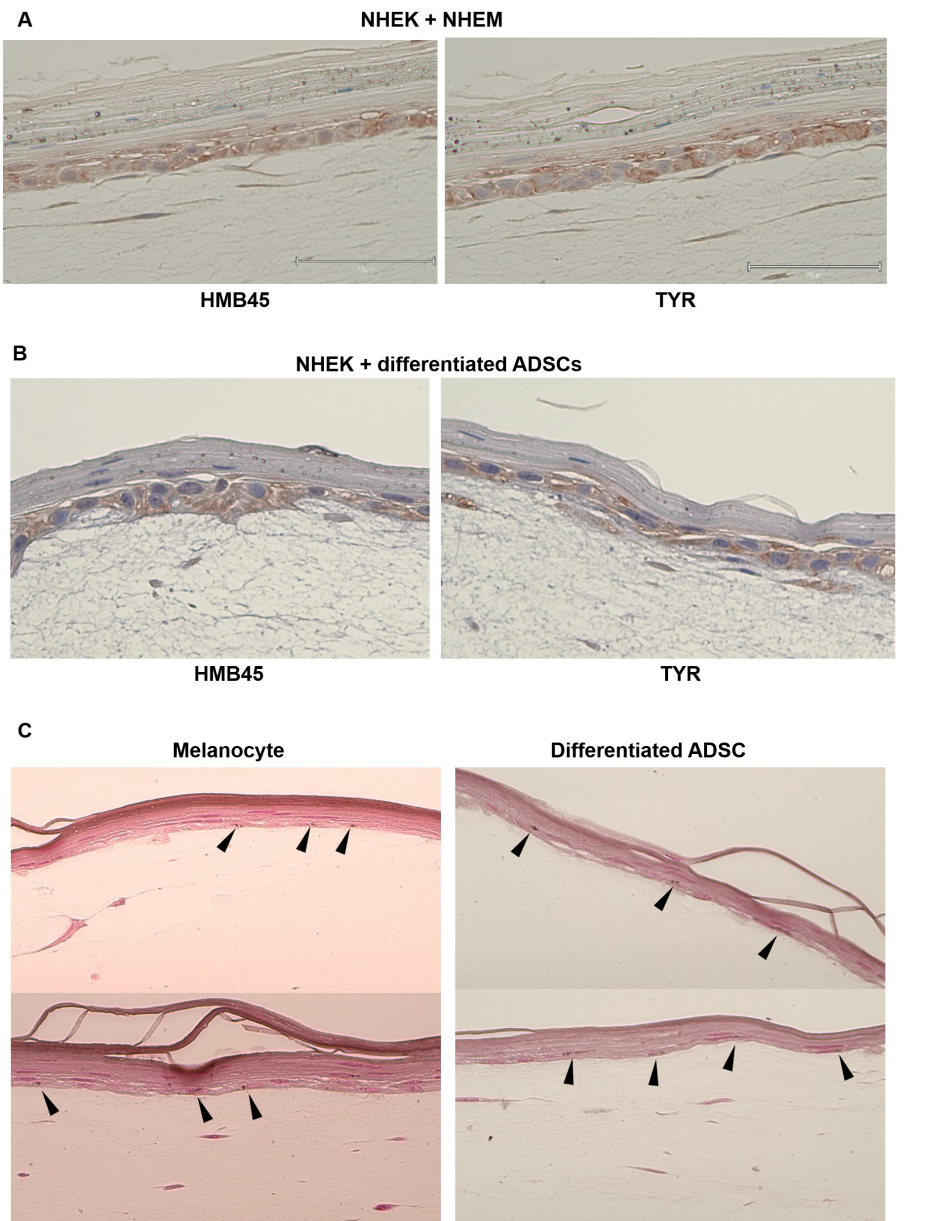
Three-dimensional epidermal culture using normal human epidermal keratinocytes (NHEKs) and normal human epidermal melanocytes (NHEMs) and NHEKs and differentiated ADSCs. HMB45- and tyrosinase (TYR)-positive cells were observed in the basal layer of both epidermis-like structures (A). Melanin deposits were induced by UVB irradiation in 3D epidermal culture using NHEKs and NHEMs, as well as in NHEKs and differentiated ADSCs and were detected by Fontana-Masson staining (B).

3D models (NHEKs + NHEMs or NHEKs + differentiated ADSCs) showed cells positive for tyrosinase and HMB45 at the basal layers of the 3D epidermis, which mimics normal human epidermis (Fig 8A and 8B).

### Melanin production of the 3D epidermal model by NHEKs + NHEMs or NHEKs + differentiated ADSCs (Fontana-Masson staining)

In addition, melanin deposits were induced in 3D models (NHEKs + NHEMs or NHEKs + differentiated ADSCs) by UVB irradiation (Fig 8C).

## Discussion

Melanocytes are melanin-producing cells found in the skin, hair follicles, eyes, inner ear, bones, heart and brain of humans [17–19] and play an indispensable role in the pigmentation of skin and its appendages. Additionally, melanocyte stem cells are thought to be derived from neural crest cells and to reside in the epidermis of the skin and hair follicles [15]. Therefore, many researchers have focused on melanocyte stem cells and stem cell niches in hair follicles and the interfollicular epidermis [20]. After melanoblasts enter the lateral pathway, they migrate subectodermally at the same time as the dermatome undergoes an epithelial-to-mesenchymal transition into the dermis and then enter the epidermis and hair follicles [8]. This suggests that some melanocyte progenitors could remain and reside in subcutaneous adipose tissue during embryogenesis. The data obtained in this study and our previous finding of keratinocyte progenitors residing in ADSCs strongly support this possibility.

In this study, we first examined the expression of HMB45 in undifferentiated ADSCs because HMB45 is an antibody that recognizes a melanoma- and melanocyte-specific antigen [13, 14] that is expressed in amelanotic malignant melanoma [21]. HMB45 reacts with sialylated PMEL17 in the fibrillar matrix in early melanosomes [11]. As expected, the expression of HMB45 was observed in undifferentiated ADSCs by immunofluorescence microscopy (Fig 1), RT-PCR (Fig 2), western blotting (Fig 4A), and immunoelectron microscopy (Fig 5), and the constitutive expression of HMB45 in undifferentiated ADSCs was completely diminished by siRNA (Fig 3). The expression of HMB45 was increased in differentiated ADSCs, as shown by immunofluorescence microscopy. Immunoelectron microscopy confirmed that increased immunoreactivity of HMB45 was observed in membrane-bound structures resembling those in melanocytes.

We also tested the expression of microphthalmia-associated transcription factor (MITF) because MITF is considered a master gene for cells of the melanocyte lineage [12]. We found that MITF was expressed in undifferentiated ADSCs by immunofluorescence microscopy (Fig 1), RT-PCR (Fig 2), and western blotting (Fig 4) and that the expression level of MITF was higher in differentiated ADSCs than in undifferentiated ADSCs. Differentiated ADSCs also expressed other melanocytic markers (Melan-A, MATP, MelEM, Mel2, except for TYR) at variable levels, and these expression levels in differentiated ADSCs were much higher than those in undifferentiated ADSCs, as determined by immunofluorescence microscopy (Fig 1). Additionally, the expression of KIT and PAX3, which are expressed in melanocytes, was found to be elevated in differentiated ADSCs, as determined by RT-PCR (Fig 2). These results were observed in all ADSCs from Caucasian, African and Asian individuals (S1A, S1B, S2A and S2B Figs in S1 File). Moreover, pigmented L-DOPA-positive cells were observed among differentiated ADSCs and NHEMs (Fig 7A). These observations indicate that melanocyte progenitor cells reside in adipose tissue and that these progenitor cells might have the potential to differentiate into mature melanocytes.

We also considered whether there would be a specific subset of ADSCs that have the ability to differentiate into melanocytes. Briefly, we sorted ADSCs with anti-Mel2 antibodies, which are antibodies against melanocytic membrane protein markers. Specifically, Mel2-positive cells expressed increased levels of melanocytic markers (HMB45 and MITF) according to RT-PCR. This result also indicates that there are progenitor melanocyte lineage cells in ADSCs, and these progenitors could be a main subset that differentiate into mature melanocytes.

Ito et al. reported de novo hair follicle regeneration in adult mouse skin after large and deep wounding (reaching the subcutaneous tissue). The regenerated hair follicles establish a stem cell population, express known molecular markers of follicle differentiation, produce a hair shaft and progress through all stages of the hair follicle cycle [22]. We expected, at least in part, that melanocyte progenitors residing in subcutaneous tissue could contribute to this “de novo” regeneration of hair follicles. We also expected that the so-called pluripotent stem cells in undifferentiated ADSCs could differentiate into mature melanocytes during culture with melanocyte-specific media. To demonstrate this possibility in the future, researchers could perform single-cell analysis [23] of melanocyte progenitor cells or another type of ADSC.

Keratinocyte and melanocyte progenitors reside in human subcutaneous adipose tissue [9, 10, and the present study], and keratinocyte and melanocyte stem cells are thought to be derived from neural crest cells and to migrate to the epidermis of the skin and hair follicles [18]. For the migration of keratinocytes and melanocyte stem cells, at least 2 routes from the neural crest to the skin have been speculated: one route is through the surface side of the skin and another route is through the subcutaneous tissue along with vessels and neurons. Yoshida et al. reported that there might be 4 distinct stages of melanocyte differentiation, and the first step might be migration to the dermis, which composes the upper part of subcutaneous fat tissue. The second step is the stage before epidermal entry, and the third step involves cell proliferation after entering the epidermal layer. The last step is integration into the developing hair follicles [24].

The presence of melanocyte progenitors in subcutaneous adipose tissue might support the possibility that melanocyte stem cells (melanoblasts) migrate to subcutaneous tissue, reach the dermis, and then enter the epidermis and hair follicles. Additional studies using MITF-eGFP mice should be performed in the future.

In addition, both 3D models (NHEKs + NHEMs or NHEKs + differentiated ASCs) showed cells positive for tyrosinase and HMB45 in the basal layers of the 3D epidermis, which mimicked normal human epidermis (Fig 8A and 8B). Furthermore, melanin deposits were observed by Fontana-Masson staining (Fig 8C). These findings indicate that differentiated ADSCs could act as mature melanocytes in 3D epidermal models.

This study demonstrates that melanocyte progenitor cells reside in human subcutaneous adipose tissue, suggesting that these melanocyte and keratinocyte progenitors residing in human subcutaneous tissue could be used for skin diseases and skin rejuvenation in the future.

## Conclusion

These results demonstrate that melanocyte progenitor cells reside in human subcutaneous adipose tissue and that these cells might have the potential to differentiate into mature melanocytes. Melanocyte and keratinocyte progenitors residing in human subcutaneous tissue will be available for use in skin diseases and skin rejuvenation in the future.

## Supporting information

S1 File (DOCX)
