# Supplementary material for: Melanocyte progenitor cells reside in human subcutaneous adipose tissue
Source: PLoS One. 2021 Aug 25;16(8):e0256622. doi: 10.1371/journal.pone.0256622 (PMC8386863; doi:10.1371/journal.pone.0256622)
Supplement: S1 File — (DOCX) [file pone.0256622.s001.docx]

**S1 File**

**Cells and culture**

Human adipose-derived stem cells(ADSCs); African (0000550179, 20TL063594) and Asian (20TL027210) #PT-5006; Lonza) were used in this study. ADSCs were cultured (passages 2-6) with melanocyte medium (Dermo Life Basal Medium, Lifeline) for 7-14 days, and these cells were designated “differentiated ADSCs”. Normal human epidermal melanocytes (NHEMs from an infant African American, CAT# KM-4009, Kurabo) were used as positive controls.


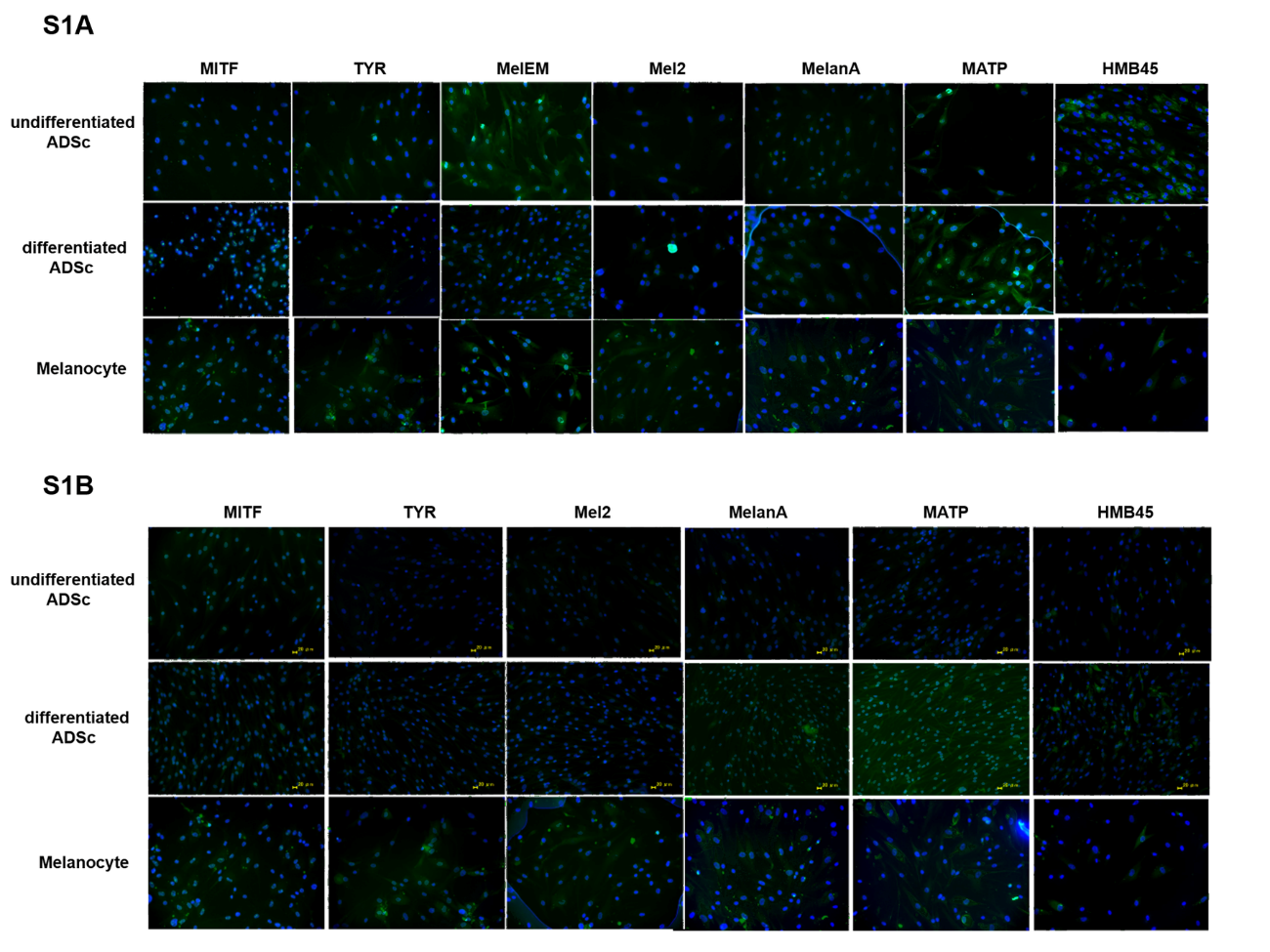


**S1 Fig. Immunofluorescence microscopy of African and Asian ADSCs.**

The expression levels of MelanA, MATP, HMB45, LAMP1 MITF, MelEM, and Mel2 in undifferentiated and differentiated African ADSCs, as well as melanocytes, were examined by immunofluorescence microscopy (A). The expression levels of MelanA, MATP, HMB45, LAMP1 MITF, and Mel2 in undifferentiated and differentiated Asian ADSCs, as well as melanocytes, were examined by immunofluorescence microscopy (B).The expression of these melanocytic markers was observed in ADSCs from African and Asian individuals.


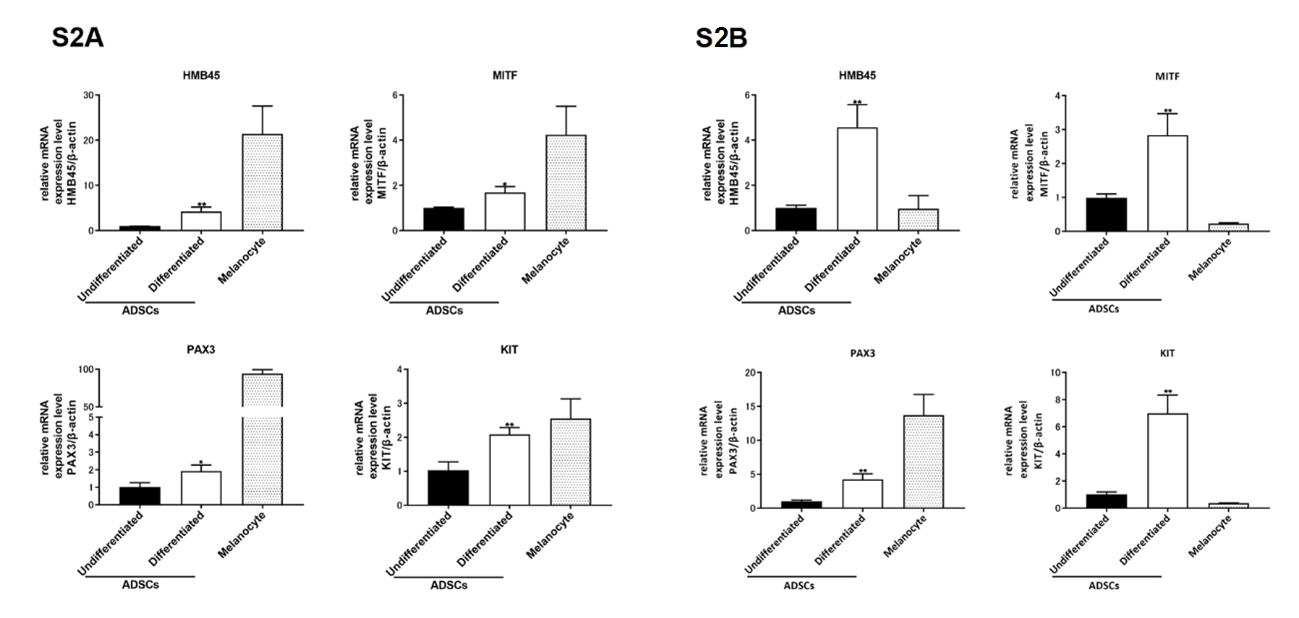


**S2 Fig. RT-PCR of African and Asian ADSCs.**

The expression levels of HMB45, MITF, PAX3, and KIT in undifferentiated African ADSCs and differentiated African ADSCs were compared with those in melanoma cells by RT-PCR (A). The expression levels of HMB45, MITF, PAX3, and KIT were higher in differentiated African ADSCs than in undifferentiated African ADSCs. The expression levels of HMB45, MITF, PAX3, and KIT in undifferentiated Asian ADSCs and differentiated Asian ADSCs were compared with those in melanoma cells by RT-PCR (B). The expression levels of HMB45, MITF, PAX3, and KIT were higher in differentiated Asian ADSCs than in undifferentiated Asian ADSCs. The expression of these melanocytic markers was observed in ADSCs from African and Asian individuals.


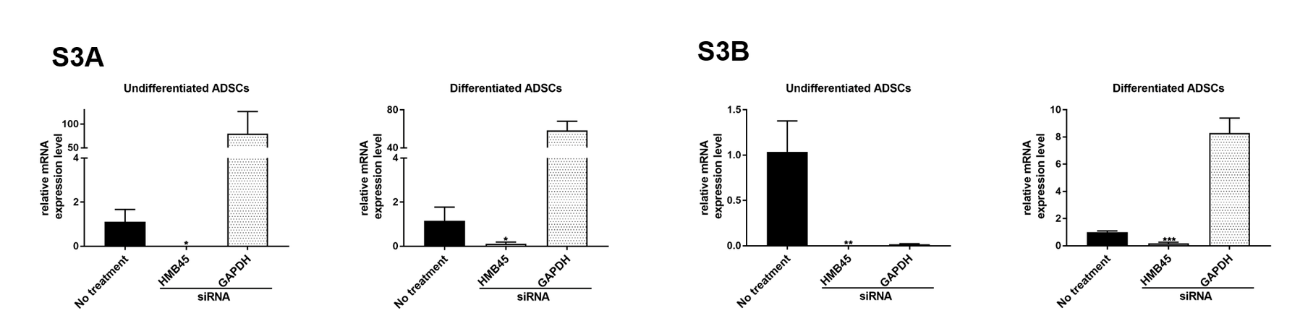


**S3 Fig. siRNA of African and Asian ADSCs.**

siRNA resulted in the downregulation of HMB45 in undifferentiated African ADSCs and differentiated African ADSCs (A). siRNA resulted in the downregulation of HMB45 in undifferentiated Asian ADSCs and differentiated Asian ADSCs (B). Downregulation of HMB45 was observed in ADSCs from African and Asian individuals.
